# Supplementary material for: Working with Young People at Risk of Suicidal Behaviour and Self-Harm: A Qualitative Study of Australian General Practitioners’ Perspectives
Source: Int J Environ Res Public Health. 2021 Dec 8;18(24):12926. doi: 10.3390/ijerph182412926 (PMC8701929; doi:10.3390/ijerph182412926)
Supplement: Supplementary file 1 [file ijerph-18-12926-s001.zip › Supplementary File 1_COREQ Checklist.pdf]

Supplementary File 1

COREQ Checklist

A checklist of items that should be included in reports of qualitative research. You must report the page number in your manuscript where you consider each of the items listed in this checklist. If you have not included this information, either revise your manuscript accordingly before submitting or note N/A.

Topic	Item No.	Guide Questions/Description	Reported on Page No.
**Domain 1: Research team and reflexivity**			
Personal characteristics			
Interviewer/facilitator	1	Which author/s conducted the interview or focus group?<br><br>J.R., Y.P., S.J.B., and A.L.<br><br>Of the eight interviews/group interviews conducted, seven of these were facilitated primarily by the senior researcher on the study (J.R.), with secondary assistance from another senior researcher (Y.P.) and a junior researcher (S.J.B.). The other individual interview was facilitated by another senior researcher on the study (A.L.), who has a similar skill set and research training to J.R.	4
Credentials	2	What were the researcher’s credentials? E.g. PhD, MD.<br><br>J.R. – BSc (Hons), MSc Applied Psy, PhD<br>Y.P. – BPsych (Hons), MPsych (Clin), PhD<br>S.J.B. – BA (Hons), PGCert Stats<br>A.L. – BSc (Hons), MClin Neuropsych, PhD	N/A
Occupation	3	What was their occupation at the time of the study?<br><br>J.R. – Associate Professor<br>Y.P. – Research Fellow, Clinical Psychologist<br>S.J.B. – Research Assistant<br>A.L. – Research Fellow	N/A
Gender	4	Was the researcher male or female?<br><br>All interviewers were female.	N/A
Experience and training	5	What experience or training did the researcher have?<br><br>All interviewers were researchers with psychology-related backgrounds and qualifications, who had previous experience in qualitative interviewing, and a range of professional experience conducting research in healthcare systems with both practitioners and young people, as well as on the topic of suicide and self-harm.	4
Relationship with participants			
Relationship established	6	Was a relationship established prior to study commencement?<br><br>Interviewers were unknown to participants prior to the commencement of the study and recruitment.	N/A

Participant knowledge of the interviewer	7	What did the participants know about the researcher? E.g. personal goals, reasons for doing the research.<br><br>Interviewers introduced themselves at beginning of the interviews, and explained their roles, occupations, and purpose of the research.	N/A
Interviewer characteristics	8	What characteristics were reported about the interviewer/facilitator? E.g. bias, assumptions, reasons and interests in the research topic.<br><br>Not applicable.	N/A
**Domain 2: Study design**			
Theoretical framework			
Methodological orientation and theory	9	What methodological orientation was stated to underpin the study? E.g. grounded theory, discourse analysis, ethnography, phenomenology, content analysis.<br><br>The theoretical framework and orientation informing data collection and analysis was an essentialist/realist, experiential, inductive approach. Reflexive thematic analysis approaches described by Braun and Clarke (2006, 2012, 2019, 2020) were used to analyse the data.	3, 4
Participant selection			
Sampling	10	How were participants selected? E.g. purposive, convenience, consecutive, snowball sampling.<br><br>GP participants were identified via their clinics which were purposively sampled. A range of clinics in the recruitment region were selected to be approached, differing by clinic size, type, and location, in order to enhance the likelihood of a diverse range of GPs taking part with differing experiences. Once clinics were selected, GPs from those clinics were invited to take part in the study and self-selected to participate.	3
Method of approach	11	How were participants approached? E.g. face-to-face, telephone, mail, email.<br><br>Clinics were identified and contacted directly about the study via email and telephone, with assistance from the WA Primary Health Alliance (WAPHA), which operates Western Australia's Primary Health Networks (PHN's). Interested GPs from those clinics were invited to take part. Participants then elected to participate in either an individual interview or a group interview with other participating colleagues from their clinic.	3
Sample size	12	How many participants were in the study?<br><br>Fifteen.	3
Non-participation	13	How many people refused to participate or dropped out? Reasons?<br><br>There were no withdrawals from the study; those who expressed interest took part in either an individual or group interview.	N/A
Setting			
Setting of data collection	14	Where was the data collected? E.g. home, clinic, workplace.	4

		Interviews were undertaken at the GPs' clinics in the Perth and Perth South Primary Health Network catchment regions of Western Australia.	
Presence of nonparticipants	15	Was anyone else present besides the participants and researchers?<br><br>In two of the individual interviews (P5 and P6) one additional person was present (a clinical psychologist in one, and another researcher not associated with this study in another). Any contributions these external parties made to the interviews were removed from the analysis, although it is possible that the presence of these external parties may have contributed to the content of participants' responses.	N/A
Description of sample	16	What are the important characteristics of the sample? E.g. demographic data, date.<br><br>Nine participants identified as male, and six as female. Further demographic data were collected from eight participants as the other seven participants failed to complete the demographic questionnaire and did not respond to follow-up. Of the eight who completed the demographic form, the mean age was 45.25 years (*SD* = 6.45; range: 37-53 years). English was the primary language spoken at home for six of the eight GPs, and none identified as Aboriginal or Torres Strait Islander. Four participants practiced primarily in suburban locations, two in metropolitan locations, one in both suburban and metropolitan locations, and one in a regional location. The average duration of professional practice for participants was 12.63 years (*SD* = 10.14; range: 2-30).	3
Data collection			
Interview guide	17	Were questions, prompts, guides provided by the authors? Was it pilot tested?<br><br>The interview schedule is outlined in Supplementary File 2. It was developed by the research team by consulting the previous literature and identifying research gaps and important questions to ask GPs. The schedule was not piloted before the study.	3
Repeat interviews	18	Were repeat interviews carried out? If yes, how many?<br><br>No repeat interviews were conducted.	N/A
Audio/visual recording	19	Did the research use audio or visual recording to collect the data?<br><br>Yes, all interviews were audio-recorded with consent from participants.	4
Field notes	20	Were field notes made during and/or after the interview or focus group?<br><br>Yes, notes were taken during and after the interviews to identify key topics and ideas. Thematic maps and memos in the form of digital and paper notes were also created during the data analysis process to help with theme generation and refinement.	4
Duration	21	What was the duration of the interviews or focus group?<br><br>Interviews ranged from 15 to 53 minutes (*M* = 37 minutes).	4

Data saturation	22	Was data saturation discussed?<br><br>In line with reflexive thematic analysis approaches where the usefulness of the concept of saturation has been questioned (Braun and Clarke, 2021), we decided that saturation was not best suited for this study. Instead, we followed the guidelines of information power recommended by Malterud et al. (2016) – that is, that the data were sufficiently rich and novel to address the aim of the study, to support the analysis, and to generate new understandings.	3
Transcripts returned	23	Were transcripts returned to participants for comment and/or correction?<br><br>No.	N/A
**Domain 3: Analysis and findings**			
Data analysis			
Number of data coders	24	How many data coders coded the data?<br><br>One (I.B.W.), who conducted data/transcript coding and initial theme generation and structure development.	4
Description of the coding tree	25	Did authors provide a description of the coding tree?<br><br>Not applicable.	N/A
Derivation of themes	26	Were themes identified in advance or derived from the data?<br><br>Derived from the data (i.e., inductive approach).	3
Software	27	What software, if applicable, was used to manage the data?<br><br>NVivo 12 (QSR International Pty Ltd., 2018) was used to store and manage the qualitative data. An online whiteboard tool Miro (https://miro.com/) assisted with the creation of thematic maps and theme structuring.	4
Participant checking	28	Did participants provide feedback on the findings?<br><br>No.	N/A
Reporting			
Quotations presented	29	Were participant quotations presented to illustrate the themes/findings? Was each quotation identified? E.g. participant number.<br><br>Yes, all quotations were identified by interview number. As there were multiple participants in the group interviews, we were not always able to provide specific participant numbers for these participants, as there were instances where this could not be determined from the transcripts and audio-data. Instead, those in the group interviews are instead identified by gender, with an additional specifying number wherever possible. All findings were illustrated with relevant quotations.	4, 4-11
Data and findings consistent	30	Was there consistency between the data presented and the findings?<br><br>Yes, all data was interpreted in the Discussion section in relation to existing literature and novel findings.	4-14
Clarity of major themes	31	Were major themes clearly presented in the findings?<br><br>Yes, we reported sever major themes.	4, 4-11

Clarity of minor themes	32	Is there a description of diverse cases or discussion of minor themes?<br><br>Disconfirming case analysis was conducted throughout the analysis process to identify and account for data that was discrepant to the themes and patterns identified. Overall, GPs' views were quite congruent, although at times they were contradictory, and discrepancies were reported as part of the properties of each relevant main theme. Additionally, minor themes were not exemplified as 'subthemes' – rather they were reported and discussed as being part of the properties of each major theme.	4, 4-11

Developed from: Tong A, Sainsbury P, Craig J. Consolidated criteria for reporting qualitative research (COREQ): a 32-item checklist for interviews and focus groups. *International Journal for Quality in Health Care*. 2007. Volume 19, Number 6: pp. 349 – 357
