# Supplementary material for: Working with Young People at Risk of Suicidal Behaviour and Self-Harm: A Qualitative Study of Australian General Practitioners’ Perspectives
Source: Int J Environ Res Public Health. 2021 Dec 8;18(24):12926. doi: 10.3390/ijerph182412926 (PMC8701929; doi:10.3390/ijerph182412926)
Supplement: Supplementary file 1 [file ijerph-18-12926-s001.zip › Supplementary File 2_Interview Schedule for General Practitioners.pdf]

Supplementary File 2

Interview Schedule for Qualitative Consultations with General Practitioners

1) Have you recently or in the past had to manage any young people at risk of suicide?

2) How would you distinguish between young people seriously considering suicide, and those mildly depressed and not suicidal?
    a. *What type of assessment do you engage in when you see a patient who has self-harmed or has suicidal ideation; do you use a standardised instrument and/or a clinical assessment?*

3) If you see a patient who is showing signs of depression, has self-harmed or has suicidal ideation, what are your next steps? E.g., do you/does your practice have standardised protocols/standardised measures that you are required to follow?

4) What, if any, are some of the challenges you experience when engaging with a young person who is showing signs of depression, has self-harmed, or has suicidal ideation?

5) What do you think is best practice when undertaking an assessment of depression and suicidality in young people?

6) What would be the barriers to implementing this approach in your practice?

7) What kind of training have you attended in the past to manage young people at risk of suicide?
    a. *What was your experience of this training? What could be improved?*

8) What kind of training or resources do you need to improve your confidence and/or ability to undertake a 'gold standard' assessment of depression and suicidality in young people?

9) What are the most appropriate methods for delivering training on depression and suicide risk assessment to GPs?

a. *What type of setting? How long do you think the training should be? How frequently? Booster sessions after the main training session? What incentives would influence GPs to attend?*
